# Volunteers’ growth mindset and continuance intention: what are the roles of nostalgia and positive emotions?

**DOI:** 10.3389/fpsyg.2023.1169221

**Published:** 2023-11-03

**Authors:** Heetae Cho, Hyun-Woo Lee, Taehee Kim

**Affiliations:** ^1^Department of Sport Science, Sungkyunkwan University, Suwon, Republic of Korea; ^2^Department of Physical Education and Sports Science, National Institute of Education, Nanyang Technological University, Singapore, Singapore; ^3^Department of Kinesiology and Sport Management, Texas A&M University, College Station, TX, United States

**Keywords:** growth mindset theory, nostalgia, positive emotions, continuance intention, volunteer

## Abstract

This study examined the cognitive and affective aspects of volunteering experiences by focusing on the relationships between volunteers’ growth mindset, nostalgia, positive emotions, and their intention to continue volunteering. A total of 364 responses were collected from volunteers who had volunteered within the past 5 years. Results showed that the growth mindset had a positive effect on nostalgia, which in turn positively affected valenced emotions toward volunteering. Nostalgia and volunteers’ positive emotions positively influenced their intention to continue volunteering. All indirect effects via nostalgia were significant. This study lays the groundwork to identify the role of nostalgia in volunteerism and contributes to extending the literature on growth mindset and mindset theory.

## Introduction

Volunteers are individuals who believe that volunteerism is a means to develop their character (Wilson and Musick, 1999). Since volunteers do not rely on any monetary compensation, organizations have to explore different ways to encourage continuous volunteer participation (Garner and Garner, 2011). Consequently, retaining volunteers has been an ongoing struggle for organizations. In academic fields, researchers have investigated various factors for maintaining volunteers, such as volunteer management (Cho et al., 2020b), volunteer satisfaction (Pauline, 2011), volunteer engagement (Vecina et al., 2012, 2013), and brand heritage (Curran et al., 2016). Previous research also examined the role of positive emotions in the context of prosocial behavior (Jiménez and Fuertes, 2005; Cavanaugh et al., 2015; Aknin et al., 2018). Although these findings provide valuable insight for organizations to implement strategies to convince volunteers to continue their service, volunteers’ internal mindset or growth perspective has not received much attention. Specifically, there has been limited research on the growth mindset in the context of volunteerism (Han et al., 2018). Thus, this study attempts to explore volunteers’ inner aspects that may influence their intention to continue volunteering.

Complementarily, some individuals believe that their abilities can be sharpened through efforts to learn and practice; this is known as a growth mindset (Dweck, 2008). When volunteers have growth mindsets, they may continuously seek ways to volunteer, despite the challenges that may arise. In line with this reasoning, as individuals with growth mindsets do not perceive challenges to be an indication of a limit to their character development (Dweck, 2000; Yeager and Dweck, 2012), volunteers’ resilience and a positive outlook on volunteerism can protect them from negative emotions and facilitate the experience of positive emotions. Hence, volunteers with a growth mindset are likely to have higher continuance intentions, as they perceive such opportunities as a platform to learn skills and enhance their personal growth. In addition, the positive outlook fostered by this mindset encourages individuals to seek opportunities for self-improvement. Given the critical role of a growth mindset in individuals’ behavioral outcomes, studies on growth mindset were conducted across various fields, including education (Dweck, 2015; Brougham and Kashubeck-West, 2017; Yeager et al., 2019), organizational psychology (Visser, 2013), and tourism (Lee et al., 2018). The common finding of these previous studies is that individuals can develop a growth mindset when their focus is on learning and helping, which does not rely on external rewards. In this respect, a growth mindset can play a pivotal role in volunteering intention. Despite the apparent usefulness of the growth mindset in different fields (Visser, 2013; Brougham and Kashubeck-West, 2017; Lee et al., 2018; Yeager et al., 2019), it has not been extensively studied in the context of volunteerism.

Besides examining a cognitive factor (i.e., growth mindset), this study also investigates emotional factors to better understand volunteers’ continuance intention. Researchers noted that individuals’ emotions are critical to understanding individuals’ behavior (Bagozzi et al., 2000; Baumeister et al., 2007). In particular, past research highlighted that the experience of positive emotions facilitates approach behavior (Bagozzi and Pieters, 1998; Koenig-Lewis and Palmer, 2014). In other words, individuals’ positive emotions act as a signal for them to continue to engage in the behavior. Positive emotion is an umbrella term that encompasses a wide range of emotions, such as joy, pleasure, happiness, and hope; among them, nostalgia has garnered a growing recognition for its complex features (Wildschut et al., 2006; Sedikides et al., 2008; Barrett et al., 2010; Puente-Díaz and Cavazos-Arroyo, 2021; Santini et al., 2023). Initially known as a symptom of a medical condition (Hofer, 1934), nostalgia has advanced over the years to be labeled as a bittersweet emotion. The bitterness represents the yearning to relive a memory, while the sweetness represents the fondness of looking back at the memory (Davis, 1979). Recently, most researchers have come to the consensus that nostalgia is a predominantly positive emotion, as this emotion is derived from positive memories and experiences, which can further generate positive emotions in the individuals’ present selves (Wildschut et al., 2011; Cho, 2023). The role of nostalgia has been identified in various fields, such as consumerism (Hwang and Hyun, 2013; Chen et al., 2014; Fan et al., 2020; Gong et al., 2023), tourism (Leong et al., 2015; Cho et al., 2019), and organizational psychology (Leunissen et al., 2018). However, volunteers’ nostalgia and its relationship with a growth mindset remain limited.

Unlike most behaviors, volunteering is associated with an individual’s emotional responses that are derived from their personal beliefs (Jiménez and Fuertes, 2005). Given that a growth mindset is a set of beliefs unique to an individual, it is logical to assume that volunteers’ growth mindsets and emotions play a critical role in their behavioral outcomes. However, while mindset theory has been used to explain individuals’ beliefs and achievements (e.g., Dweck, 2008; Hochanadel and Finamore, 2015), empirical studies have not been conducted in the context of the cognitive and affective aspects of volunteers. One of the reasons why this gap exists is because there was a limited theoretical approach to capturing volunteers’ affective aspects. Thus, as day in and day out there is increasing attention to the importance of the roles of volunteers for a successful sport event, it is imperative to delve into their cognitive and affective factors with rigorous methodology. As such, this study recognized the potential for applying this concept to understanding volunteerism and proposed the idea of testing how a growth mindset and emotions work closely together to influence volunteers’ intention to continue volunteering. Specifically, we elucidated this psychological process based on mindset theory and highlighted the significance of volunteers’ perceptions and emotions toward volunteerism and their effects on their behavioral responses. In particular, we underscored the role of volunteer nostalgia as a predominantly positive emotion derived from positive memories and experiences, which can further generate positive emotions in individuals’ present selves (Wildschut et al., 2011; Leong et al., 2015; Cho et al., 2019). Hence, this study provides an alternative to existing research by examining how volunteers’ growth mindsets influence nostalgia and positive emotions, which might contribute to their intention to continue volunteering. The findings of this study contribute to the theoretical advancement of the role of a growth mindset and nostalgia in volunteerism. From a managerial standpoint, the findings of this study provide insight into unique strategies that can enhance individuals’ intentions to continue volunteering.

## Theoretical background and hypothesis development

### Mindset theory

Mindset theory, developed by Dweck (2008), is a theory of motivation that illustrates individuals’ goal orientation as determined by their beliefs about the malleability of their abilities and intelligence. This theory proposes that mindsets can be classified into two extremes: the fixed mindset and the growth mindset. The former carries an implicit belief that an individual’s talents and abilities are fixed and not flexible to change (Dweck, 2008). Individuals with fixed mindsets desire to appear intelligent, which makes them more likely to shun challenges and give up easily. Believing that their potential to enhance their intelligence and abilities is fixed, individuals with this mindset perceive efforts to enhance those qualities as futile, and their inability to see these self-imposed restrictions causes them to ignore useful criticism. Thus, these individuals can believe they do not have control over their potential and feel threatened by others’ successes, leading them to achieve less than their full potential (Dweck, 2008).

On the other hand, growth mindsets refer to individuals’ beliefs that their talents and abilities are assets with the potential to be developed and grow to fruition through effort and practice (Murphy and Dweck, 2016). Hence, the growth mindset instills a passion for learning in individuals, which increases their propensity to embrace challenges and display resilience in the face of setbacks (Yeager and Dweck, 2012; Hochanadel and Finamore, 2015). Instead of perceiving effort to be futile, these individuals perceive it to be a path to mastery, and with the belief that learning can sharpen their abilities and intelligence, they are open to criticism (Dweck, 2008). In short, growth mindsets hold various positive psychological benefits, allowing individuals to reach higher levels of achievement. Dweck (2015) further noted that, while effort is key to attaining individuals’ goals, they still need to push themselves to explore new strategies and seek feedback from others to grow, with the caveat that when individuals have a pessimistic view of their limited abilities, they experience negative emotions, such as anxiety, and might fail to achieve their desired outcomes.

Mindset theory is frequently applied to understand learning within the context of education (e.g., Dweck, 2009; Brougham and Kashubeck-West, 2017). Other studies have extended the applicability of mindset theory to better understand leaders, coaches (Heslin and Keating, 2017), and consumers (Murphy and Dweck, 2016). Researchers found that mindset theory is applicable in the context of occupation, and a growth mindset is a useful concept for management settings (Caniëls et al., 2018). While there is existing research on the role of the growth mindset in the context of occupational psychology, there is a dearth of knowledge on another important human resource: unpaid volunteers. Specifically, the role of a growth mindset as a cognitive factor affecting volunteers’ emotional experiences has not yet received scholarly attention.

Initially known only as a symptom of a medical condition (Hofer, 1688), scholars have expanded nostalgia as a bittersweet emotion over the years, with the meaning broadened and conceptualized as a sentimental longing for the past (Davis, 1979; Cho et al., 2014). Nostalgia influences employees’ positive behavior in an occupational setting by increasing their work meaning (Leunissen et al., 2018). Additionally, nostalgia can combat psychological threats (Juhl et al., 2010). In a similar manner, empirical evidence demonstrates that individuals with growth mindsets develop emotional regulation strategies that help them buffer against negative emotions and broader psychological threats (Schroder et al., 2017). Furthermore, researchers have noted that individuals’ growth mindsets have positive relationships with positively-valenced emotions (Hwang et al., 2019). Thus, a growth mindset is a belief or a cognitive factor (Mueller and Dweck, 1998), while nostalgia can be understood as an emotional factor (Wildschut et al., 2006; Sedikides et al., 2008).

Given that nostalgia is a predominantly positive emotion (Routledge et al., 2013a; Cho and Chiu, 2020; Cho, 2023), it is possible that having a growth mindset can generate nostalgia. Specifically, volunteers with growth mindsets reflect on situations that have contributed to their talents and abilities. Hence, their continuous desire to grow and learn may make them reflect on experiences that have contributed to their learning process. These experiences have the potential to give rise to nostalgia as they are considered positive experiences, and volunteers may yearn to acquire similar learning opportunities again. Based on this understanding, we postulate the following hypothesis:

*H1*: Volunteers’ growth mindset positively affects nostalgia.

Next, this study explored the relationship between a growth mindset and positive emotions. Positive emotions refer to subjective experiences that involve positive valence, including but not limited to joy, pride, and interest (Fredrickson, 2001). Tugade and Fredrickson (2004) highlighted that resilient individuals recover from stressful experiences within a short period of time and rebound from stressful experiences using positive emotions. This indicates that individuals with growth mindsets embrace challenges, which nurtures them to be resilient in the face of setbacks (Yeager and Dweck, 2012; Hochanadel and Finamore, 2015). Recently, Zarrinabadi et al. (2022) and Intasao and Hao (2018) found that growth mindset positively relates to and plays a critical role in enhancing positive emotions. Howell (2016) also recognized the importance of one’s mindset in affecting their experience of positive and negative emotions. Consistent with these findings, Garland et al. (2010) found that more resilient individuals have the potential to self-generate positive emotions. This is because they worry less about possibly negative future consequences and focus on the present, allowing them to recover faster. The findings discussed above indicate that resilience in individuals facilitates the self-generation of positive emotions. Recent empirical research also supports the relationship between volunteers’ growth mindset and positive emotion in an educational context (Daniels et al., 2022). Therefore, the current study proposes the following hypothesis:

*H2*: Volunteers’ growth mindset positively affects positive emotions.

In various academic fields, a growth mindset has been positively associated with individuals’ behavioral intentions (Brougham and Kashubeck-West, 2017; Han et al., 2018; Orvidas et al., 2018). For example, in the context of education, researchers have provided empirical evidence that a growth mindset positively affects students’ academic performance (Dweck, 2009; Brougham and Kashubeck-West, 2017). For example, Sarrasin et al. (2018) found that the promotion of students’ growth mindset through the teaching of neuroplasticity has been shown to have a positive and significant influence on their academic motivation, academic achievement, and brain activity. Moreover, in sport psychology, according to Orvidas et al. (2018), mindsets regarding fitness were found to have a significant impact on how frequently individuals engage in exercise. Specifically, individuals having stronger growth mindset toward fitness are more likely to involve exercise regularly and have high exercise intention (Orvidas et al., 2018). In the field of volunteerism, Han et al. (2018) highlighted that a moral growth mindset is a source of motivation for voluntary service engagement. This implies that when individuals perceive their moral character to be malleable and improvable through effort, this increases their drive to be more involved in such services and opportunities. These findings suggest that individuals with growth mindsets create a drive to engage in positive behavior, allowing them to grow. Similarly, this study proposes that volunteers’ growth mindsets increase their intention to continue volunteering, as they perceive volunteering as a platform that drives them to be better individuals, thereby motivating them to continue. Lee et al.’s (2021) research also supports the aforementioned mechanism. Therefore, based on the above discussion, we hypothesize that:

*H3*: Volunteers’ growth mindset positively affects intentions to continue volunteering.

### Nostalgia and positive emotions

Nostalgia consists of positive and negative feelings and has a unique nature (Cho et al., 2014, 2021b). Specifically, nostalgic feelings possess positive emotion that arises from recalling positive past experiences, while it is also associated with negative emotions as individuals are aware that their positive experiences cannot be relived (Davis, 1979). However, the portion of positive emotion is much greater than the part of negative emotion. Thus, we consider it a predominantly positive emotion (Sedikides et al., 2004; Cho, 2023). Previous studies found that nostalgia could produce positive psychological outcomes (Routledge et al., 2008; Cho et al., 2021b). For instance, Wildschut et al. (2011) mentioned that nostalgia could generate positive emotions in the present self. Existing findings further noted that it acts as a repository for positive affect, and individuals who recalled a nostalgic experience reported a higher level of positive affect than those who recalled an ordinary experience (Wildschut et al., 2006). The many psychological benefits of nostalgic experiences are the generation of positive affect and the maintenance of psychological fortitude by managing potential psychological threats (Sedikides et al., 2008). Experiencing nostalgia also increases positive affect and facilitates terror management (Routledge et al., 2008). In addition, an increase in positive affect via nostalgia heightens individuals’ psychological health (Routledge et al., 2013b). Another positive consequence of nostalgia is that it provides meaning in life and bridges one’s past, present, and future identities (Sedikides et al., 2008). In the context of volunteerism, when individuals recall a personal volunteer experience that is positively-valenced and feel nostalgic, the emotions generate further positive emotions in the present self. In addition, this would enable individuals to bridge their identity as volunteers and encourage their continued involvement in volunteerism. Hence, the present study follows the reasoning that volunteers’ nostalgia would positively affect existing positive emotions and proposes the following hypothesis:

*H4*: Volunteers’ nostalgia positively affects positive emotions.

### Continuance intention

Understanding volunteers’ intentions to continue volunteering is crucial for businesses that rely heavily on them. Thus, researchers have investigated diverse factors that influence volunteers’ intention to continue volunteering, such as volunteer management (Cho et al., 2020b), volunteer engagement (Vecina et al., 2012), and volunteer satisfaction (Pauline, 2011). Among the diverse antecedents of individuals’ behavioral outcomes, we focused on the role of volunteers’ nostalgia as a predominantly positive emotion (Cho and Chiu, 2020). Nostalgia has been recognized as a significant factor in understanding individuals’ behavioral responses in various fields (Cho, 2020; Juhl et al., 2020). For instance, in the field of consumerism, Chen et al. (2014) illustrated the role of nostalgia in consumers’ intention to eat at nostalgic-themed restaurants. Hwang and Hyun (2013) also found that the experience of nostalgic feelings has a positive effect on intentions to revisit luxury restaurants, suggesting that the experience of nostalgia creates an attraction even before the actual experience and instills their intention to continue visiting restaurants based on past experiences. The experience of nostalgia also influences charity intentions, such as prosocial behavior, as it generates a higher level of emotion toward charity behaviors (Li, 2015). In line with this, the present study hypothesized that volunteers’ nostalgia would elicit a recall of previous positive volunteer experiences, which would positively affect their intention to continue volunteering.

*H5*: Volunteers’ nostalgia positively affects intention to continue volunteering.

The last relationship explored in this study was the effect of volunteers’ positive emotions on their intention to continue volunteering. Early findings by Bagozzi and Pieters (1998) on goal-directed behavior highlight that positive emotions are associated with the continuation of a current behavior that leads individuals to attain their personal goals. Namkung and Jang (2010) also explained the relationship between positive affect and behavioral intentions by emphasizing that individuals use their affect as a basis for their judgment. This makes them lean toward favorable behavior when experiencing positive emotions. Consistent with this, existing findings show that positive emotions can positively influence customer behavioral intentions, such as intentions to revisit (Kincaid et al., 2010; Prayag et al., 2015). Additionally, in an occupational setting, Staw et al. (1994) noted that employees who experienced positive emotions at work were more likely to engage in favorable work behaviors, allowing them to achieve their goals. More relevant to the present study’s context, Aknin et al. (2018) and Cho and Joo (2022) stressed that positive emotions from prosocial behaviors act as reinforcement and encourage individuals to continue engaging in prosocial behavior. Therefore, this study hypothesized that the intention to continue volunteering, a type of prosocial behavior, is influenced by the experience of positive emotions:

*H6*: Volunteers’ positive emotions positively affect intention to continue volunteering.

## Materials and methods

### Participants and procedures

Individuals who have volunteer experiences were recruited to participate in this study. Specifically, participants included undergraduate and postgraduate students, staff, and visitors to universities, who had volunteered within the past 5 years. This research sets a time frame of 5 years to ensure that they can still remember their volunteering experiences to provide credible responses (Cho et al., 2017). Data were collected through convenience sampling at the benches and drop-off points of four universities in a Southeast Asian city-state. Research assistants collected data at places where many people gathered in the universities, and it was confirmed whether respondents had volunteer experiences within the last 5 years before starting a survey. Another reason why we chose certain universities was due to the increasing attention in terms of voluntary management in the city. The survey was approved by the institutional review board of the Southeast Asian university. A total of 452 responses were collected for this study (a response rate of 85.2%). A total of 88 responses, which were less than 50 percent completed, were excluded. This study conducted a *t*-test and compared the sample means of the data from both response (85.2%) and non-response (14.8%) to identify non-response bias. According to the results, there was no significant difference between the two responses, confirming that there was no non-response bias. In addition, to identify common method bias, we conducted a single-factor test and confirmed that the value exceeded 50% (Harman, 1960). As a result, this study used 364 volunteers, consisting of 174 (47.8%) males and 190 (52.2%) females. The average age was 24.23 years old (SD = 7.3). The sample consisted of participants from different races (i.e., 294 Chinese, 80.8%; 27 Indian, 7.4%; 26 Malay, 7.1%), educational backgrounds (i.e., 183 A level or below, 50.3%; 82 with diploma, 22.5%; 69 with university degree, 19%; 20 with graduate degree, 5.5%), and income levels (i.e., 147 under $5,000, 42.6%; 144 from $5,000 to $9,999, 21.4%; 84 $10,000 or over, 23.1%). Most of them (66.1%) volunteered for less than 5 years in a non-profit organization. About half (54.1%) of the participants have volunteered in the last 3 years. An average participant (78%) volunteered fewer than six times in the past 3 years. The majority of the respondents were single (93.4%).

### Measures

The questionnaire consisted of five sections: mindsets, nostalgia, positive emotions induced by volunteering, intentions to continue volunteering, and background information. To measure growth mindset, four items were borrowed from the Implicate Persons Theory Measure (Levy et al., 1998), which showed acceptable reliability and validity in previous research (Park and John, 2012). Volunteers’ nostalgia was measured using the Volunteer Nostalgia Scale (Cho and Lee, 2021), consisting of 20 items across five subfactors: volunteer experience, volunteer environment, socialization, personal identity, and group identity. Positive emotion toward volunteering was measured using 10 items (i.e., attentive, interested, alert, excited, enthusiastic, inspired, proud, determined, strong, and active) of the Positive and Negative Affect Schedule (PANAS) scale (Watson et al., 1988). Intention to continue volunteering was measured using three items adapted from Hoye et al. (2008). The scales used in this study showed reliability and validity in previous research (e.g., Crawford and Henry, 2004; Park and John, 2012; Cho and Joo, 2022), and the items are in the form of 7-point Likert scales, where participants indicate their level of agreement from (1) strongly disagree to (7) strongly agree. The wording of these items is listed in Table 1, and psychometric properties are reported in Table 2.

**Table 1:** Descriptive statistics of measures.

Item and construct	*M*	SD	Skewness	Kurtosis
**Growth mindset (** Levy et at., 1998 **)**				
(GM1) I can change even my most basic qualities	3.97	1.95	−0.09	−0.56
(GM2) I can significantly change my basic characteristics	3.89	2.09	0.16	−0.82
(GM3) I can substantially change the kind of person who I am	4.24	2.22	−0.14	−0.81
(GM4) No matter what kind of person I am, I can always change very much	3.95	2.10	0.14	−0.67
**Positive emotions (** Watson et al., 1988 **)**				
(PE1) Attentive	5.42	1.22	−0.98	1.66
(PE2) Interested	5.49	1.21	−1.20	2.33
(PE3) Alert	5.46	1.26	−1.00	1.75
(PE4) Excited	5.40	1.42	−0.94	1.23
(PE5) Enthusiastic	5.42	1.43	−0.96	1.39
(PE6) Inspired	5.30	1.80	−0.89	0.60
(PE7) Proud	5.17	1.86	−0.74	0.45
(PE8) Determined	5.38	1.47	−0.83	0.97
(PE9) Strong	5.22	1.59	−0.73	0.64
(PE10) Active	5.41	1.53	−0.86	0.94
**Volunteer experience (** Cho and Lee, 2021 **)**				
(EX1) Remembering volunteer experience that I enjoyed	5.36	1.60	−0.93	0.78
(EX2) The atmosphere of the event that I attended	5.30	1.57	−0.76	0.51
(EX3) Remembering the moment of learning volunteer knowledge	5.07	1.65	−0.83	0.87
**Volunteer environment (** Cho and Lee, 2021 **)**				
(EN1) The size of the place I volunteered	4.62	2.86	−0.54	−0.50
(EN2) The equipment I used during the volunteer activity	4.62	2.59	−0.52	−0.43
(EN3) The architectural design of the event I volunteered	4.56	2.63	−0.48	−0.46
(EN4) The apparels that I received	4.67	2.93	−0.57	−0.56
(EN5) Memorable weather during the volunteer activity	4.50	2.82	−0.52	−0.56
**Personal identity (** **Cho and Lee, 2021** **)**				
(PI1) Sense of accomplishment as a volunteer	5.48	1.57	−1.15	1.74
(PI2) My value as a volunteer	5.25	1.63	−0.89	0.84
(PI3) A feeling of achievement during the volunteer activity	5.39	1.56	−0.81	0.53
(PI4) Positive feelings about myself as a volunteer during the volunteer activity	5.25	1.71	−0.87	0.76
(PI5) Pride in being a volunteer of the event that I attended	5.12	1.77	−0.76	0.43
**Group identity (** Cho and Lee, 2021 **)**				
(GI1) Unique characteristics of the volunteer social group	4.95	2.01	−0.57	−0.14
(GI2) The traditions of the volunteer group	4.59	2.30	−0.23	−0.71
(GI3) Volunteer group rituals at the event	4.46	2.45	−0.19	−0.79
**Socialization (** Cho and Lee, 2021 **)**				
(SO1) Memories of meeting new volunteers	5.19	1.82	−0.84	0.60
(SO2) Memories of socializing with others during the volunteer activity	5.34	1.64	−0.86	1.01
(SO3) Memories of getting useful information by talking to others during the volunteer activity	5.06	2.00	−0.65	0.14
(SO4) Memories of building friendships with other volunteers during the volunteer activity	5.37	1.65	−0.96	1.09
**Behavioral intention (** Hoye et al., 2008 **)**				
(BI1) It is likely that I will engage in volunteer work within the next 3 years	5.41	2.42	−1.00	0.49
(BI2) I intend to engage in volunteer work within the next 3 years	5.38	2.45	−0.94	0.30
(BI3) I plan to engage in volunteer work within the next 3 years	5.23	2.60	−0.74	−0.13

**Table 2:** Psychometric properties and factor correlations of scales.

								Φ				
Item and construct	λ	S.E.	ρ	AVE	1	2	3	4	5	6	7	8
1. Growth mindset			0.89	0.68	**0.83**	0.21	0.20	0.19	0.15	0.32	0.24	0.12
GM1	0.80	0.02										
GM2	0.91	0.02										
GM3	0.76	0.03										
GM4	0.82	0.02										
2. Positive emotions			0.95	0.65		**0.80**	0.82	0.52	0.76	0.59	0.68	0.53
PE1	0.72	0.03										
PE2	0.81	0.02										
PE3	0.75	0.03										
PE4	0.79	0.02										
PE5	0.77	0.02										
PE6	0.78	0.02										
PE7	0.79	0.02										
PE8	0.91	0.01										
PE9	0.85	0.02										
PE10	0.87	0.02										
3. Volunteer experience			0.82	0.61			**0.78**	0.71	0.88	0.71	0.84	0.58
EX1	0.80	0.02										
EX2	0.76	0.03										
EX3	0.78	0.02										
4. Volunteer environment			0.92	0.70				**0.84**	0.62	0.60	0.64	0.24
EN1	0.87	0.02										
EN2	0.88	0.02										
EN3	0.89	0.01										
EN4	0.77	0.02										
EN5	0.77	0.02										
5. Personal identity			0.92	0.71					**0.84**	0.65	0.83	0.48
PI1	0.87	0.02										
PI2	0.86	0.02										
PI3	0.80	0.02										
PI4	0.85	0.02										
PI5	0.82	0.02										
6. Group identity			0.82	0.61						**0.78**	0.71	0.35
GI1	0.80	0.02										
GI2	0.94	0.01										
GI3	0.91	0.01										
7. Socialization			0.91	0.73							**0.85**	0.45
SO1	0.90	0.01										
SO2	0.90	0.01										
SO3	0.81	0.02										
SO4	0.80	0.02										
8. Behavioral intention			0.97	0.91								**0.96**
BI1	0.93	0.01										
BI2	0.99	0.00										
BI3	0.95	0.01										

All factor loadings are significant (*p* < 0.001). The square root of AVEs bolded in diagonals of the correlation matrix.

### Data analysis

Confirmatory factor analysis (CFA) was used through a statistical tool, Mplus, to assess the psychometric properties of the measurement model. Following the guidelines of Fornell and Larcker (1981) and Anderson and Gerbing (1988), factor loadings, composite reliability, and convergent and discriminant validity were examined using average variance extracted (AVE) and factor correlations. We followed Hu and Bentler’s (1999) criteria to assess the model fit. Absolute fit indices of the Standardized Root Mean Square Residual (SRMR; Bentler, 1990), Root Mean Square Error of Approximation (RMSEA; Steiger, 1990), and incremental Comparative Fit Index (CFI; Bentler, 1990) were used. A second-order CFA was conducted to fit nostalgia as a representative higher-order factor of the five sub-constructs. Structural equation modeling was used to test the hypothesized relationships. A bias-corrected bootstrapping estimation with 1,000 iterations was used to examine all indirect effects.

## Results

### Measurement model

The descriptive statistics of all the measures are presented in Table 1. The data were deemed usable because skewness between −2 and 2 and kurtosis between −7 and 7 of the items indicated a fairly normal distribution (Finney and DiStefano, 2013). The results of the CFA indicated a good fit of the data (χ^2^ = 1565.555, df = 593, CFI = 0.926, SRMR = 0.045, RMSEA = 0.067). As shown in Table 2, the factor loadings ranged from 0.72 to 0.99, and the composite reliability values ranged from 0.82 to 0.97, indicating acceptable internal consistency (α > 0.70). In addition, AVE values ranged from 0.61 to 0.91, showing acceptable convergent validity (AVE > 0.50). Next, we assessed the discriminant validity of the measurement model. According to the results, all square roots of AVE values were larger than the respective factor correlations, except for three correlations (volunteer experience-positive emotion, volunteer experience-volunteer personal identity, and volunteer experience-volunteer socialization) (Table 2). Consequently, we conducted a chi-square difference test to provide additional evidence of discriminant validity. The chi-square difference test, with models constraining the corresponding factor correlation as 1, yielded significant results (volunteer experience-positive emotion: Δχ^2^ = 96.52; volunteer experience-volunteer personal identity: Δχ^2^ = 48.31; volunteer experience-volunteer socialization: Δχ^2^ = 71.19; all *p*s < 0.001 at df = 1), indicating acceptable discriminant validity.

### Structural model

**Figure 1:**
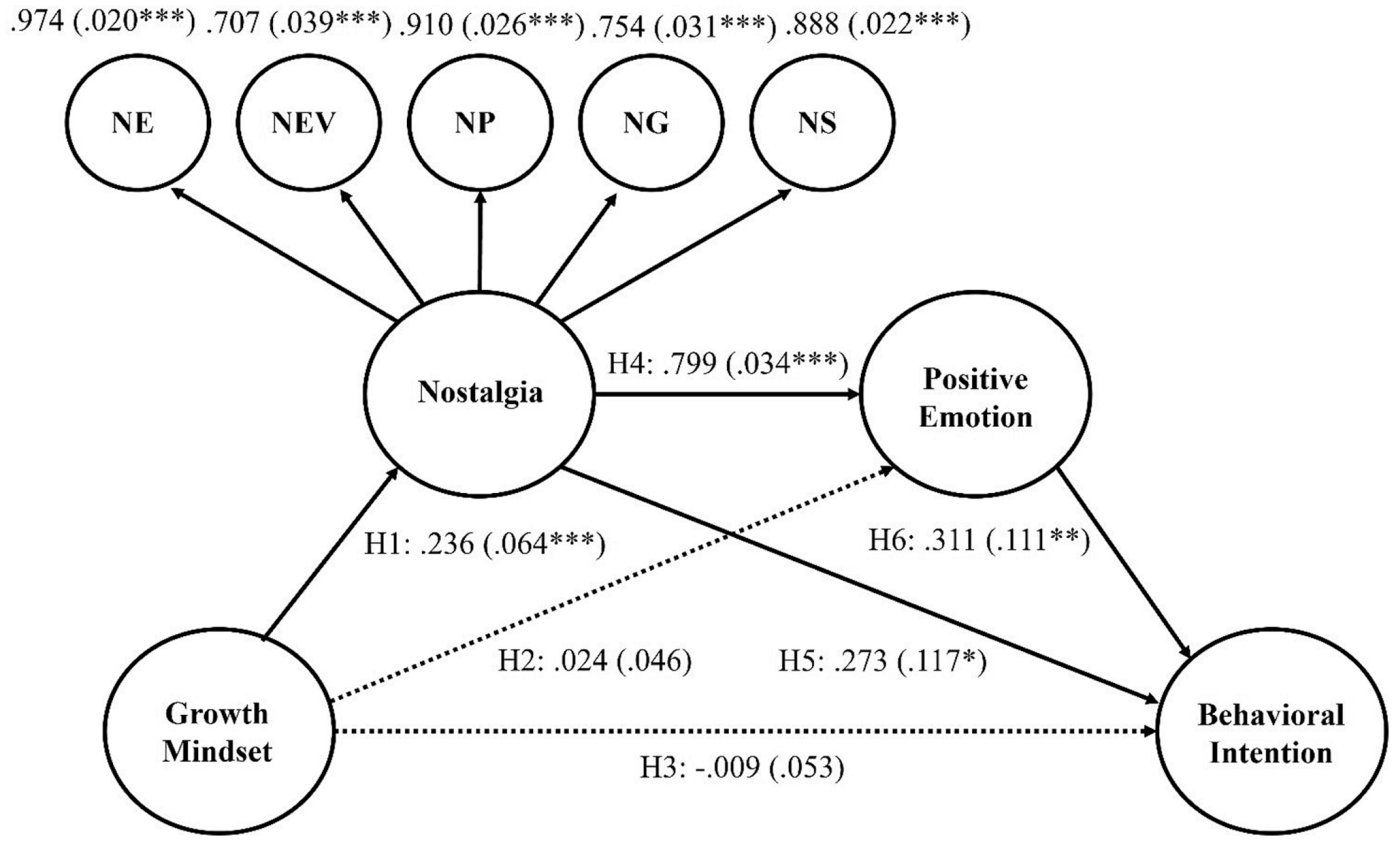
Standardized coefficients of research model. Dotted lines are nonsignificant paths. Nostalgia is a higher-order factor of volunteer experience, volunteer environment, personal identity, group identity, and socialization. Standard errors in parentheses. *p < 0.05. **p < 0.01. ***p < 0.001.

The structural model and equivalent second-order nostalgia factor model indicated a good fit for the data (χ^2^ = 1641.703, *df* = 610, CFI = 0.921, SRMR = 0.052, RMSEA = 0.068). Factor loadings of first-order factors on second-order factor nostalgia were all significant with values above 0.70 (volunteer experience: λ = 0.97; volunteer environment: λ = 0.71; personal identity: λ = 0.91; group identity: λ = 0.75; socialization: λ = 0.89; all *p*s < 0.001). As shown in Figure 1, H1, H4, and H5 were supported, whereas H2 and H3 were not supported by direct effects. Therefore, in addition to the primary focus, this study examined the indirect effects in the research model and identified the significant indirect effects, as reported in Table 3. The central role of nostalgia is illuminated, as every direct and indirect path via this construct was significant, demonstrating the largest coefficient effect sizes.

**Table 3:** Bootstrap results of mediated paths.

Mediated paths	Standardized	Unstandardized	BC Interval
**1. Growth mindset ➔ Behavioral intention**			
Total effect	0.121*	0.156	(0.019, 0.219)
Total indirect effect	0.130**	0.168	(0.072, 0.202)
Via positive emotion	0.007	0.010	(−0.013, 0.036)
Via nostalgia	0.064*	0.083	(0.018, 0.129)
Via nostalgia and positive emotion	0.059*	0.075	(0.022, 0.121)
Direct effect	−0.009	−0.012	(−0.098, 0.075)
**2. Growth mindset ➔ Positive emotion**			
Total effect	0.212***	0.150	(0.115, 0.310)
Total indirect effect (via nostalgia)	0.189***	0.133	(0.100, 0.274)
Direct effect	0.024	0.017	(−0.049, 0.110)
**3. Nostalgia ➔ Behavioral intention**			
Total effect	0.522***	0.773	(0.430, 0.605)
Total indirect effect (via positive emotion)	0.249**	0.368	(0.116, 0.422)
Direct effect	0.273*	0.405	(0.071, 0.443)

Reported BC intervals are the bias-corrected 95% confidence interval of standardized estimates. *p < 0.05. **p < 0.01. ***p < 0.001.

## Discussion

The present study examined the relationships among volunteers’ growth mindset, positive emotions, nostalgia, and intention to continue volunteering. Testing the hypothesized model on the empirical data supported the essential roles of a growth mindset and nostalgia in volunteering intentions. Of the six hypothesized relationships, four were found to be statistically significant. First, this study found that a growth mindset had a positive effect on nostalgia (H1). Consistent with the appraisal theory of emotions (Lazarus, 1991, 2001), this study identified a cognitive component affecting an emotional component, that is, a growth mindset induces positive emotion through nostalgia. Dweck (2015) found that a growth mindset leads to higher self-esteem, which can elicit nostalgia; however, the causal directions are still unclear, as some evidence suggests that nostalgia can also boost self-esteem (Sedikides et al., 2016), which directs attention toward positive personal experiences (Sedikides et al., 2008; Zhou et al., 2012). These findings imply that growth mindset causes a positive cascading effect on an individual’s thoughts about themselves, thus generating nostalgia.

The second finding of this study was that there was no significant direct relationship between volunteers’ growth mindset and positive emotions toward volunteering (H2). Previous studies have asserted a strong connection between resilient individuals and the generation of positive emotions (Tugade and Fredrickson, 2004), which is in line with the concept of a growth mindset, as individuals with this mindset perceive setbacks as challenges that allow them to improve themselves; thus, transforming them into resilient individuals (Yeager and Dweck, 2012; Hochanadel and Finamore, 2015). This result can be attributed to the substantial influence of volunteer nostalgia in the relationship between a growth mindset and positive emotions. This study found that nostalgia had a positive effect on positive emotions (H4), indicating full mediation; there was an indirect effect of a growth mindset on positive emotions through nostalgia. As nostalgia is an emotion derived from positive experiences and memories, it is reasonable to assume that when a positive past experience is brought to individuals’ focal attention, they are reminded of the positive emotions embedded in that experience. This creates a carryover effect on the present by enabling them to experience positive emotions in their present selves (Wildschut et al., 2011). Likewise, researchers in the field of psychology found that the experience of nostalgia generated positive emotions (Wildschut et al., 2006, 2011). That is, the presence of nostalgia that arises from a growth mindset influences the experience of positive emotions, indicating the significant role of nostalgia in the cognition and emotion processes. Another speculation on why there was no direct relationship between volunteers’ growth mindset and positive emotions can be explained by social exchange theory (Cropanzano and Mitchell, 2005). Regardless of their motivation to volunteer, volunteers tend to expect advantages when contributing to their duties. Thus, they do not merely make efforts to their duties, rather volunteers’ intentions to continue may depend on fulfilling volunteers’ expectations that organizations can provide. While past findings established a connection between positive emotions and the byproducts of the growth mindset, such as resilience and self-efficacy, the findings of this study did not reveal any significant positive effects of volunteers’ growth mindset on positive emotions.

We found that both emotion-related constructs (i.e., volunteers’ nostalgia and positive emotions regarding volunteering) had direct effects on their intention to continue volunteering (H5 and H6). Also, it was found that nostalgia showed indirect effects on behavioral intention. Previous studies have explored the role of nostalgia and found the direct and indirect effects on behavioral intentions in different contexts, such as consumer behavior (Hwang and Hyun, 2013; Chen et al., 2014), sport psychology (Roslan and Cho, 2022), sports marketing (Cho and Chiu, 2020; Cho et al., 2021b), tourism (Leong et al., 2015; Cho et al., 2020a), and leisure (Cho, 2020, 2021; Cho et al., 2021a). These studies showed that nostalgia creates a sentimental longing to relive the experience and influences individuals’ decision-making directly and indirectly. Similarly, Juhl et al. (2020) noted that individuals with nostalgic feelings experienced emotions more intensely and had an improved capacity to understand others’ emotions, causing them to engage in prosocial behaviors. Moreover, Zhou et al. (2012) assert that nostalgia bolsters social connectedness and promotes the intention to help others. In line with these findings, the present study showed the positive effect of nostalgia on a specific prosocial behavior, namely, the intention to continue volunteering. Volunteers who experience nostalgia look back at their past volunteer experiences and may focus on its social aspect, increasing feelings of social connectedness. This social connectedness creates empathy, which instills an intention to continue volunteering.

Last, while no direct relationship was found between volunteers’ growth mindset and intention to continue volunteering (H3), the growth mindset was found to have an indirect relationship with the intention to continue volunteering through nostalgia and positive emotions. These findings imply the importance of the experience of emotions for individuals with positive behavioral intentions—specifically, nostalgia and positive emotions among volunteers. According to the appraisal theory of emotions (Lazarus, 1991, 2001), emotions are generated based on individuals’ cognitive appraisals of events, which in turn lead to specific reactions across different individuals. Also, Dweck (2008), who developed mindset theory, noted that individuals with a growth mindset are more likely to view challenges and failures as opportunities, and this positive appraisal of challenges and failures can lead to more adaptive emotional responses. Furthermore, these emotions shaped by their cognitive appraisal and interpretations can influence individuals’ behavior in a positive way (Lazarus, 1991, 2001). That is, the indirect effects found in this study were consistent with the appraisal theory of emotions (Lazarus, 1991, 2001); this study highlighted that the volunteers’ growth mindset was responsible for the generation of nostalgia, leading them to experience positive emotions, which might then instill their intention to continue volunteering.

### Theoretical and practical implications

The findings of this study provide a key theoretical contribution to the literature. First, the literature highlighting the potential of mindset theory (Dweck, 2008) has influenced various fields, such as education (Yeager and Dweck, 2012; Hochanadel and Finamore, 2015), occupational settings (Caniëls et al., 2018; Lee, 2018), and tourism (Lee et al., 2018; Japutra et al., 2019). Considering the fact that volunteers render their services based on their innate values instead of external rewards, such as monetary benefits, it is apparent that there is a connection between an individual’s mindset and their intention to volunteer. However, the concept of a growth mindset has not yet been understood in the context of volunteers. To address this gap, we examined volunteers’ cognitive-emotional processing and provided empirical evidence on the important role of a growth mindset in the intention to continue volunteering through the experience of emotions, extending the existing literature on growth mindset and mindset theory.

The second theoretical contribution is identifying the critical role of nostalgia in volunteerism. Nostalgia has been identified as a significant factor influencing behavioral and psychological outcomes in diverse academic fields (Hwang and Hyun, 2013; Chen et al., 2014; Leong et al., 2015; Cho et al., 2020a). In particular, nostalgia is an emotional component that plays an influential role in prosocial behavior (Zhou et al., 2012; Juhl et al., 2020) and can positively affect employees’ behavior by creating work meaning (Leunissen et al., 2018). Given that volunteerism is considered a prosocial behavior, and organizations consider volunteers as part of their human resources, it is possible to assume that volunteers’ nostalgia can enhance their behavioral responses. However, nostalgia in volunteers has not received much attention from researchers, and we attempted to address this gap by laying the necessary groundwork to identify the role of nostalgia in the context of volunteerism. In this regard, our findings can be aligned with social exchange theory (Cropanzano and Mitchell, 2005) and self-determination theory (Deci and Ryan, 1985). Volunteers commit to successful sport event management in exchange for their experiences provided by the organizations. In this mechanism, the effects of emotion and nostalgia on volunteers’ motivation to continue provided meaningful theoretical implications to the broader body of knowledge of the field.

Together with these theoretical advancements, the findings also offer practical implications by shedding light on the importance of a growth mindset among volunteers. Volunteers with growth mindsets seek to learn and improve their volunteer-related skills. They do not back down from challenges but see them as an opportunity to learn and grow. Therefore, cultivating a growth mindset in volunteers is an important point that organizations should focus on, especially considering the significant indirect effects we found. To this end, organizations can build a feedback system that provides volunteers with an assessment of their performance to help them identify their strengths and weaknesses. This system can make volunteers feel a sense of achievement and enhance their satisfaction, helping them to maintain their volunteering roles. Furthermore, organizations can offer volunteers short workshops or courses that enable them to sharpen their volunteer skills and even share sessions, so that volunteers can inspire one another.

Additionally, the results indicate the critical role of nostalgia in volunteers’ emotional and behavioral outcomes. Based on the findings of this study, organizations can attempt to evoke nostalgia according to its sub-factors. For example, organizations can look into developing programs in which there is a mix of knowledge and enjoyment to help volunteers build positive experiences. Given that environmental aspects can create nostalgic feelings, organizations can build memorable and unique designs in their venues. For socialization, practitioners can provide more opportunities for volunteers to mingle with fellow volunteers and beneficiaries of their services. Providing a photo booth for volunteers to take a picture together may be a good way to socialize with each other. If the event involves helping people directly, organizations can introduce more interactive activities between volunteers and target communities. Through this, volunteers’ work would have a personal touch, and the interaction with both fellow volunteers and the community would be etched in their hearts, increasing the likelihood of evoking nostalgia. According to the roles of personal and group identities, organizations can initiate a reward program for volunteers to feel achievement. Organizations can also affirm volunteers of their identity by giving them a certificate or plaque that serves as memorabilia for their achievement. When they look at it in the future, it can create a sense of pride. In addition, organizations can develop unique group cultures and rituals, creating bonds between volunteers.

### Limitations and future research

As with every research, this study comes with several limitations. First, to understand volunteers’ future behavior, we measured their intention to continue volunteering instead of their actual behavior. Hence, future studies could observe the relationship between volunteers’ actual and future volunteering behaviors through a longitudinal study. Second, this study only collected data from volunteers in a Southeast Asian city-state. Therefore, future studies should collect data from other countries and conduct cross-cultural research to establish external validity. In addition, this study only measured the relationships among growth mindset, nostalgia, positive emotions, and behavioral intention and did not measure the moderating role of demographic factors in the research model. Thus, we suggest that future research should consider various intermediate variables and measure how the relationships between variables can change depending on demographic factors. Finally, this study did not select a specific volunteer event to examine the relationships between the variables. Instead, respondents were asked to visualize their favorite volunteer experiences. Actual volunteer experience or even the type of volunteerism may have the potential to produce different findings on the relationships identified in this study. Therefore, future research could examine specific events and consider different types of volunteering to observe any variations.

## Data availability statement

The datasets used and/or analyzed during the current study available from the corresponding author on reasonable request.

## Ethics statement

The studies involving humans were approved by National Institute of Education, Nanyang Technological University. The studies were conducted in accordance with the local legislation and institutional requirements. The participants provided their written informed consent to participate in this study.

## Author contributions

All authors listed have made a substantial, direct, and intellectual contribution to the work and approved it for publication.
